# Ecological features of microbial community linked to stochastic and deterministic assembly processes in acid mine drainage

**DOI:** 10.1128/aem.01028-24

**Published:** 2024-12-16

**Authors:** Zhenghua Liu, Chengying Jiang, Zhuzhong Yin, Ibrahim Ahmed Ibrahim, Teng Zhang, Jing Wen, Lei Zhou, Guoping Jiang, Liangzhi Li, Zhendong Yang, Ye Huang, Zhaoyue Yang, Yabing Gu, Delong Meng, Huaqun Yin

**Affiliations:** 1 School of Minerals Processing and Bioengineering, Key Laboratory of Biometallurgy of Ministry of Education, Central South University 506609 , Changsha, China; 2 State Key Laboratory of Microbial Resources, Institute of Microbiology Chinese Academy of Sciences 85387 , Beijing, China; 3 Central Metallurgical Research and Development Institute 113311 , Cairo, Egypt; 4 Hebei Key Laboratory of Highly Efficient Exploitation and Utilization of Radioactive Mineral Resources, Ganchan, China; 5 School of Architecture and Civil Engineering, Chengdu University 74707 , Chengdu, Sichuan, China; Colorado School of Mines, Golden, Colorado, USA

**Keywords:** acid mine drainage, community assembly, species interaction, metagenome, function genes

## Abstract

Ecological processes greatly shape microbial community assembly, but the driving factors remain unclear. Here, we compiled a metagenomic data set of microbial communities from global acid mine drainage (AMD) and explored the ecological features of microbial community linked to stochastic and deterministic processes from the perspective of species niche position, interaction patterns, gene functions, and viral infection. Our results showed that dispersal limitation (DL) (48.5%~93.5%) dominated the assembly of phylogenetic bin in AMD microbial community, followed by homogeneous selection (HoS) (3.1%~39.2%), heterogeneous selection (HeS) (1.4%~22.2%), and drift (DR) (0.2%~2.7%). The dominant process of dispersal limitation was significantly influenced by niche position in temperature (*r* = −0.518, *P* = 0.007) and dissolved oxygen (*r* = 0.471, *P* = 0.015). Network stability had a significantly negative correlation with the relative importance of dispersal limitation, while it had a positive correlation with selection processes, implying changes in network properties could be mediated by ecological processes. Furthermore, we found that ecological processes were mostly related to the gene functions of energy production and conversion (C), and amino acid transport and metabolism (E). Meanwhile, our results showed that the number of proviruses and viral genes involved in arsenic (As) resistance is negatively associated with the relative importance of ecological drift in phylogenetic bin assembly, implying viral infection might weaken ecological drift. Taken together, these results highlight that ecological processes are associated with ecological features at multiple levels, providing a novel insight into microbial community assembly in extremely acidic environments.

**IMPORTANCE:**

Unraveling the forces driving community assemblage is a core issue in microbial ecology, but how ecological constraints impose stochasticity and determinism remains unknown. This study presents a comprehensive investigation to uncover the association of ecological processes with species niche position, interaction patterns, microbial metabolisms, and viral infections, which provides novel insights into community assembly in extreme environments.

## INTRODUCTION

Unraveling the ecological drivers that govern community assembly is essential yet difficult, particularly in the field of microbial ecology. Community assembly is primarily described as deterministic and stochastic processes that reflect a population’s ecological behaviors in community establishment and maintenance (1). By focusing on the deterministic processes, it is largely dependent on species niche-based interactions with other species and the environment (2). Conversely, stochastic processes are governed by ecologically similar species and primarily driven by their immigration and birth/death rates (3). These two processes interact in community assembly and could be unified into one framework, but the balancing factors of their relative importance are still unknown.

Environmental niches play a fundamental role in mediating niche-based processes during community assembly, especially for environmental selection. Species are constrained by various abiotic stresses, such as pH (1, 4), temperature (5, 6), dissolved oxygen (7, 8), and heavy metals (1, 9, 10), and their colonization probability increases with the decreasing deviation between environmental factor and relevant niche optima and the widening niche breadth termed “stress tolerance” (11). Consequently, higher environmental stresses result in greater importance of variable selection imposed by abiotic pressures. For example, determinism significantly increases and overwhelmingly governs microbial community assembly toward highly salty soil (12). Consistently, stochasticity decreases but still plays a predominant role in shaping bacterial community in highly contaminated groundwater by low pH and high heavy metals (1). In this case, it implies that the level of environmental stress could not fully explain the decreasing stochastic processes, which may be further associated with environmental niche of community. However, how niche position in environmental variables influences ecological processes in microbial community assembly is still unclear.

Species interactions serve as a biotic filtering force that governs and balances the deterministic and stochastic processes in community assembly. Theoretical models demonstrate that the balance of interaction types determines the assembly of ecological communities, and ecological selection prefers to increase the prevalence of mutualisms because mutualistic interactions could improve species persistence and community stability (13). For example, increasing specialization of mutualistic interactions between a plant and a herbivorous insect could weaken stochasticity in assembly processes as community succession (14). Competitive interactions among microbial species mediate determinism in community assembly under limited nutrient conditions, such as carbon (15) and nitrogen sources (16). These observations have led to a consensus that more deterministic processes were driven by stronger species interaction (17) and resulted in non-random structure of ecological networks (18, 19). As a consequence, deterministic processes at high salinity increase network stability and connectivity of satellite species in microeukaryotic plankton communities (18). These results show that ecological network assemblage is accompanied by deterministic and stochastic processes, while associations between network properties and ecological processes need further investigation.

Metabolisms essentially control population behaviors and drive stochastic and deterministic processes (20). For instance, stochasticity in community assembly was negatively correlated with abundance and diversity of genes involved in specialized functions (21), such as nitrogen metabolism, methane metabolism, and terpenoids and polyketides metabolism, while it was positively related to that of broad functions, such as glycolysis/gluconeogenesis and tricarboxylic acid cycle (22). Experimental evidence demonstrated that stochasticity of community assembly was raised by decreasing microbial activities in carbon metabolism, including carbohydrates, polymers, and phenolic acids (23). Consistently, the deterministic assembly process was significantly associated with some metabolites weakening autotoxin metabolism in rhizosphere microbial community (24). These findings reveal that community assembly processes are basically driven by metabolisms encompassing diverse biological processes, but metabolisms related to ecological process remain elusive.

Viruses greatly shape microbial community structure and assemblages by specific infections (25–27). According to the kill-the-winner hypothesis, viruses lyse dominant species with high cell density and release nutrients (28), which could serve as homogeneous selection (HoS) to maintain species diversity. Alternatively, the piggyback-the-winner hypothesis assumes that viruses prefer lysogenic infection of dominant species with a high growth rate (29–31), which strengthens biotic selection but weakens abiotic selection by auxiliary metabolic genes enhancing host competitiveness and environmental adaptability (32, 33). These two infection strategies are widely observed in various ecosystems and modulate community assembly, including marine (34), lake (35), hot spring (36), and acid mine drainage (AMD) (37, 38). Meanwhile, viruses could reprogram host metabolisms and redirect carbon or nitrogen cycling (34, 35, 39), which may reallocate nutrient availability for microbial species and disturb their population dynamics (40, 41). Nevertheless, how viral infection regulates stochastic and deterministic processes in community assembly is still unknown, on the basis of relevant auxiliary metabolic genes.

AMD is an extremely harsh environment with a low pH and a high concentration of heavy metals, which is an ideal model system for microbial community investigation (42). We compiled an AMD metagenomic database and explored the forces driving ecological processes and balancing stochasticity and determinism in community assembly via the method of inferring community assembly mechanisms by the phylogenetic-bin-based null model (iCAMP) (6). We aim to address the following four questions: (i) Which environmental niche optima of microbial species influence ecological processes, shaping AMD microbial community assembly, and how? (ii) How is the interaction pattern associated with ecological processes? (iii) Which biological processes are linked with the assembly processes? (iv) How do viral infections modulate stochastic and deterministic processes? We found that niche position in environmental variables of phylogenetic bin significantly constrained ecological processes and associated with global properties of interaction network. We further revealed that ecological drift was associated with the most orthologous genes (OGs), followed by dispersal limitation (DL) and specialized functions, while stochastic processes were mainly associated with broad functions. Thus, our results provide deep insights into community assembly from the perspectives of community niches, interaction patterns, and metabolisms.

## MATERIALS AND METHODS

### Sample collection, physicochemical analyses, and metagenomic sequencing

From September 2017 to November 2019, we collected 31 AMD samples including water, sediment, and biofilm from the mining area of Dabaoshan (24.52 N, 113.72 E), Mengzi (23.28 N, 103.46 E), Zijinshan (25.19 N, 116.18 E), Monyuva (22.10 N, 95.09 E), and Huaxi (26.52 N, 106.57 E). The concentration of iron (Fe^2+^ and Fe^3+^) and sulfate was measured using portable colorimeter (DR/890, HACH, USA) based on the o-phenanthroline spectrophotometry and turbidimetric method, respectively. The pH, conductivity, and temperature of each sampling site were detected by portable water quality multi-parameter meter (Star A329, Thermo Orion, USA). More details were described by a previous study (43).

Total DNA of the samples was extracted by using PowerPlant DNA isolation kit (Mo Bio Laboratories, California, USA) according to the manufacturer’s instructions, as described by a previous study (44). After qualification and quantification by Agilent 2100 Bioanalyzer and ABI StepOnePlus real-time PCR system, respectively, DNA sample libraries were sequenced on Illumina HiSeq platform at Guangdong MAGIGEN Biotechnology company (http://www.magigen.com/, Guangzhou, China).

### Public data sets of AMD metagenomes and genomes

A total of 183 AMD metagenomic samples were compiled from the National Omics Data Encyclopedia database (109) and the National Center for Biotechnology Information (74). These samples were collected from acid mine drainage environments (biofilm, sediment, soil, and acidic water) around the mining areas located in six countries including China (119), USA (31), Canada (15), Brazil (9), Germany (5), and Sweden (4). More details about the sample description and accession numbers are listed in Table S1.

### Metagenome assembly, binning, and profiling of microbial community

Raw metagenomic reads were trimmed, low-quality reads were removed by Kneaddata v0.6.1 software (45) with a quality score of “phred33,” and the retaining high-quality reads were assembled by MEGAHIT v1.2.9 software (46) with default parameters. The assembly sequences were used to reconstruct microbial genomes by metawrap v1.3.2 software (47) based on three methods with default parameters, including metaBAT2, MaxBin2, and CONCOCT. Qualities of metagenome-assembled genomes (MAGs) were accessed by CheckM software, and only MAGs with a completion of ≥70% and a contamination of ≤5% were kept for downstream analyses. All MAGs were de-replicated by dRep v3.2.2 (-sa 0.95 -pa 0.9 -nc 0.30 -comp 50 -con 10) (48). To profile the microbial species abundance, the high-quality reads of each sample were mapped to MAGs by CoverM v0.6.0 software (49) within a genome model based on rpkm (reads per kilobase per million mapped reads) method with default parameters. All the above analyses were performed by the online pipeline iMAC (https://www.biosino.org/iMAC/).

### Phylogenetic community assembly

Phylogenetic tree of MAGs was constructed by GToTree v1.7.05 software (50) based on 74 core genes. Based on the phylogenetic tree of MAGs, the algorithm of infer community assembly mechanisms by phylogenetic-bin-based null model analysis (iCAMP) (6) was applied to quantify the relative contribution of ecological processes in each phylogenetic bins during community assembly. Five ecological processes, including HoS, heterogeneous selection (HeS), homogenizing dispersal (HD), DL, and drift (DR), were identified in the iCAMP. Briefly, iCAMP first divided the MAGs into various groups based on their phylogenetic relationships (i.e., phylogenetic bins). The minimum members of one phylogenetic bin were set to 24. If the number of members in one phylogenetic bin is less than 24, these will merge into the most similar phylogenetic bin. Beta phylogenetic diversity was characterized by beta net relatedness index (βNRI) and Raup-Crick metric (RC) by using null model analysis. Five ecological processes were determined by βNRI and RC as follows: (i) βNRI < −1.96 was HoS; (ii) βNRI > 1.96 was HeS; (iii) |βNRI| ≤ 1.96 and RC < −0.95 was HD; (iv) |βNRI| ≤ 1.96 and RC > 0.95 was DL; (v) |βNRI| ≤ 1.96 and |RC| ≤ 0.95 was DR. The above analyses were conducted with “iCAMP” v1.5.12 package in R v4.2.0 (51). The phylogenetic diversity of the phylogenetic bin was calculated by “pd” function within “picante” v1.8.2 package.

### Network analyses

Based on MAG abundance, the correlation of the paired MAG was calculated by Spearman’s method, and the correlation threshold of the absolute value of Spearman’s correlation was determined by the random matrix theory (52). Only the significant correlations (*P* < 0.05) larger than the threshold were kept for network construction. For each phylogenetic bin, sub-networks were extracted by “subset_network” function on the basis of their members. Network properties were calculated by “cal_network_attr” function, including average degree, average path length, network diameter, clustering coefficient, density, heterogeneity, and centralization. Average degree is the mean number of partners linked with one node. Average path length is the mean value of the shortest distance between two nodes. Network diameter is the longest distance between two nodes. Clustering coefficient of a node is the extent of its neighbors being a complete graph. Density is the portion of actual connections to all potential connections. Heterogeneity is the variance of node degree. Centralization is the extent to which a node served as a key broker between many other nodes. Network stability is characterized by the area below the extinction curve generated from the consequences of removing a species from a network. The above analyses were performed within the packages of “microeco” v0.11.0 package (53).

According to the linkages between host and prophage, a host–prophage network was constructed. The host–prophage network was divided into sub-networks based on members of various phylogenetic bins; nestedness and modularity were calculated by “nest.smdm” function and “DIRT_LPA_wb_plus” function within “bipartite” v2.20 package, respectively.

### Function analysis of orthologous genes (OGs)

Genes of MAGs were identified by Prokka v1.14.6 (54) with default parameters. These genes were clustered into orthologous gene families by MMseqs2 v12.113e3 (55) with 70% coverage and 70% identity. Function assignment of orthologous gene families was performed by emapper v2.1.11 against eggNOG database v5.0.2 (56) through diamond method with an e-value ≤ 0.001 and a seed ortholog e-value ≤ 0.001.

### Provirus prediction, virus operational taxonomic unit (vOTU) clustering, and taxonomic assignment

Provirus sequences were identified from MAGs by PhageBoost v 0.1.7 with default parameters. All putative viral contigs were clustered into species-level vOTU at 95% ANI and 85% alignment fraction of the shorter sequence by using “aniclust.py” script from the CheckV repository (57, 58). Taxonomic assignment of vOTUs was conducted by vConTACT v2.0 (59). Briefly, a protein-sharing network based on putative virus genomes and reference genomes (ProkaryoticViralRefSeq201-Merged) via all-to-all BLASTp (60) was constructed. A pair of closely related vOTUs was grouped into a viral cluster at genus level with a similarity score of ≥1 based on the number of sharing proteins. Viral genes involved in metal resistance were identified by diamond v0.8.22.84 (61) against BacMet2 database (62).

### Statistical analyses

We calculated species niche optima for the de-replicated MAGs as the variance of environmental variable (63). Linear regression analysis was conducted by “lm” function. Correlations between relative importance of ecological processes and network properties were calculated by “cor” function via Pearson’s method. Random forest regression analysis was applied to calculate the relative importance in ecological processes for species niche breadths or network properties, which was carried out within “randomFrorest” v4.7-1.1 package. Venn diagram analyses for OGs involving various ecological processes were conducted by “ggvenn” function (64). Data visualizations were carried out by “ggplot2” v3.5.0 packages (65), and co-occurrence networks for each phylogenetic bin were displayed within “ggraph” v2.2.1.9 package (66).

## RESULTS

### Assembly of phylogenetic bins in acid mine drainage

**Fig 1:**
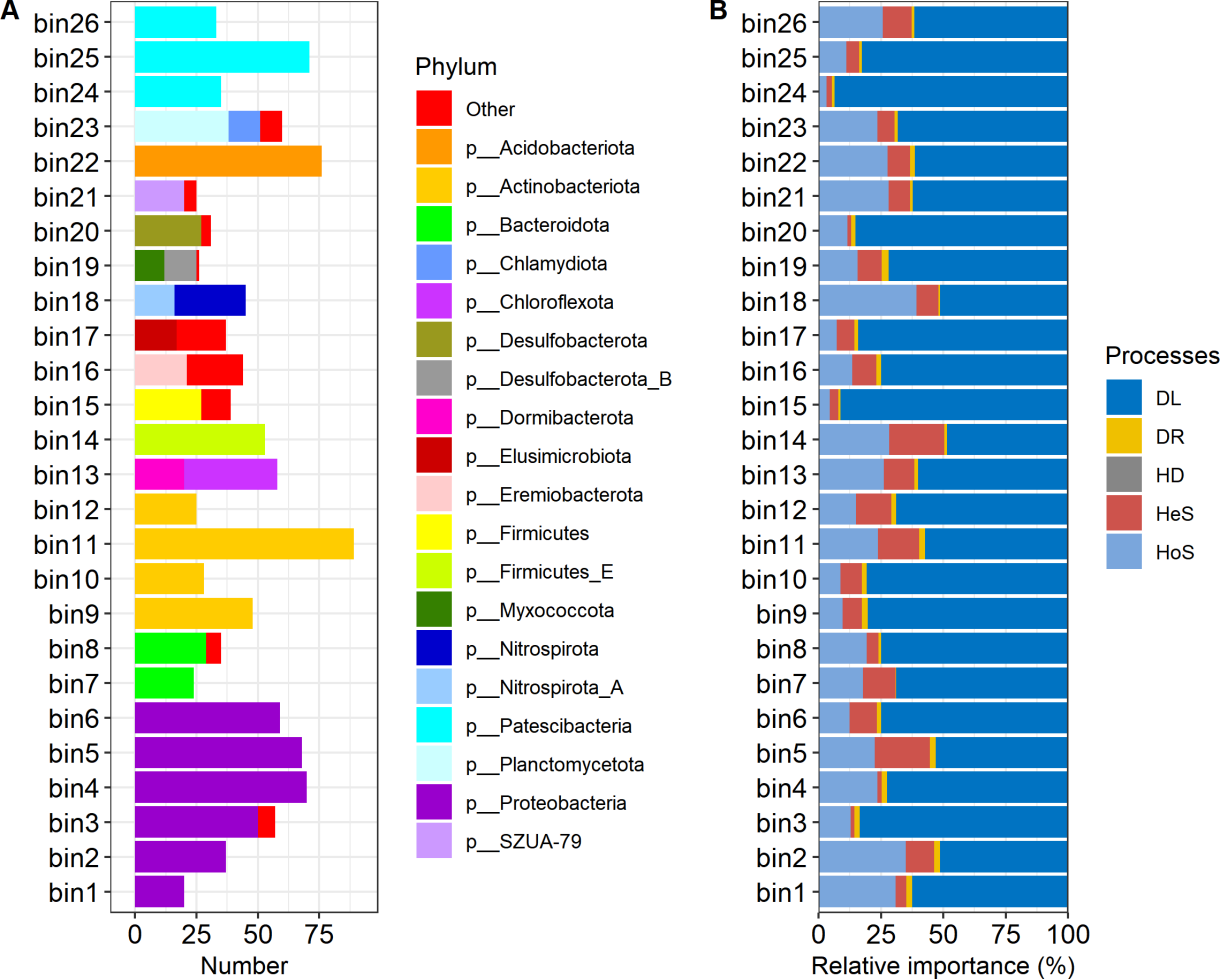
Information in phylogenetic bins consisting of phylogenetically similar species. (**A**) Members at phylum level. (**B**) Relative importance of ecological processes shaping bin assembly, including dispersal limitation (DL), drift (DR), homogeneous dispersal (HD), heterogeneous selection (HeS), and homogeneous selection (HoS).

A total of 26 phylogenetic bins were generated from 1,241 MAGs, and each phylogenetic bin included an average of 48 members, ranging from 24 to 90 (Fig. 1A). In most cases, these phylogenetic bins contained phylogenetically similar MAGs. For example, we found that 5, 4, and 3 phylogenetic bins consisted solely of Proteobacteria, Actinobacteria, and Patescibacteria, respectively. Our findings indicated that DL predominated in the assemblages of phylogenetic bins, accounting for 48.5% to 93.5% of the observed patterns (Fig. 1B). This was followed by HoS, which ranged from 3.1% to 39.2%, and HeS, which constituted 1.4% to 22.2%. DR was the least represented mechanism, contributing only 0.2% to 2.7%. Homogenizing dispersal was absent in these 26 phylogenetic bins. Linear regression analyses showed that phylogenetic diversity had no significant correlations (all *P* > 0.05) with the relative importance of ecological processes in phylogenetic bin assemblages.

### Niche position in environmental factors constrained ecological processes in the assembly of phylogenetic bins

**Fig 2:**
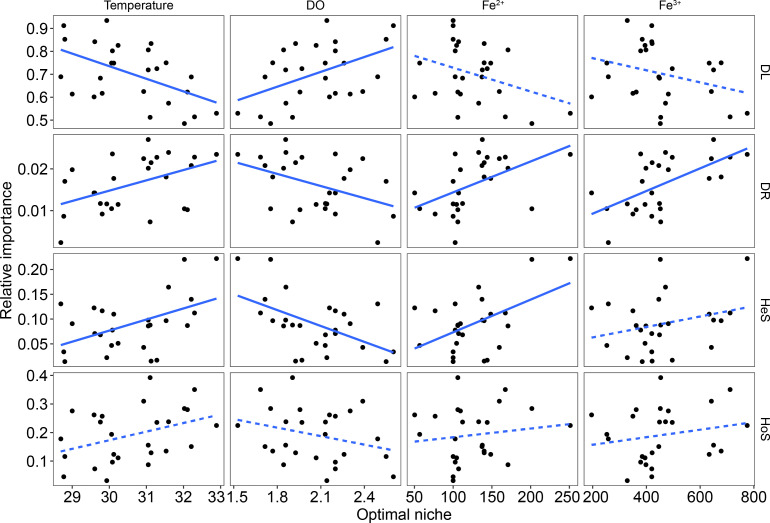
Linear relationships between relative importance of ecological processes and median environmental niches of members in phylogenetic group for temperature, dissolved oxygen (DO), and concentration of Fe^2+^ and Fe^3+^. Solid lines, *P* < 0.05; dashed lines, *P* > 0.05.

To address how niche position influences phylogenetic bin assemblage in acid mine drainage, we calculated niche optima and breadth of environmental factors for each MAG, including pH, temperature, dissolved oxygen (DO), and the concentration of Fe^2+^, Fe^3+^, Cu^2+^, and SO_4_^2-^. We found that the assembly of phylogenetic bins was significantly associated with the median niche optima of their members, specifically in terms of temperature, DO, and concentration of Fe^2+^ and Fe^3+^ (Fig. 2). For example, the niche position of temperature was negatively related to the relative importance of dispersal limitation (*P* = 0.007, R^2^ = 0.237), while it was positively related to that of drift (*P* = 0.019, R^2^ = 0.177) and heterogeneous selection (*P* = 0.013, R^2^ = 0.198). Conversely, the niche position of DO had a positive correlation with the relative importance of dispersal limitation (*P* = 0.015, R^2^ = 0.189), while it had a negative correlation with that of drift (*P* = 0.035, R^2^ = 0.294) and heterogeneous selection (*P* = 0.005, R^2^ = 0.252). Moreover, increasing the niche position of Fe^2+^ and Fe^3+^ concentration significantly enhanced the relative importance of drift. Similarly, increasing the niche position of Fe^2+^ concentration also significantly (*P* = 0.005, R^2^ = 0.169) strengthened the relative contribution of heterogeneous selection in phylogenetic bin assemblage. Results of random forest regression analyses showed that, for the stochastic processes, the niche position of Fe^3+^ and Fe^2+^ concentration played the most important roles in dispersal limitation and drift, respectively. For the deterministic processes, the niche position of DO and Fe^3+^ concentration was a primary driver of heterogeneous selection and homogeneous selection, respectively.

To assess the niche breadth of a phylogenetic bin, we calculated the coefficient of variation (CV) based on the niche optima of their MAG members. Our results showed that the relative importance of drift in phylogenetic bin assembly had a significantly negative correlation with the CV of increasing niche position of pH and SO_4_^2-^ concentration (Fig. S1). Moreover, the relative importance of heterogeneous selection was significantly decreased with the CV of the niche optima of DO (Fig. S1).

### Interaction network properties associated with ecological processes

**Fig 3:**
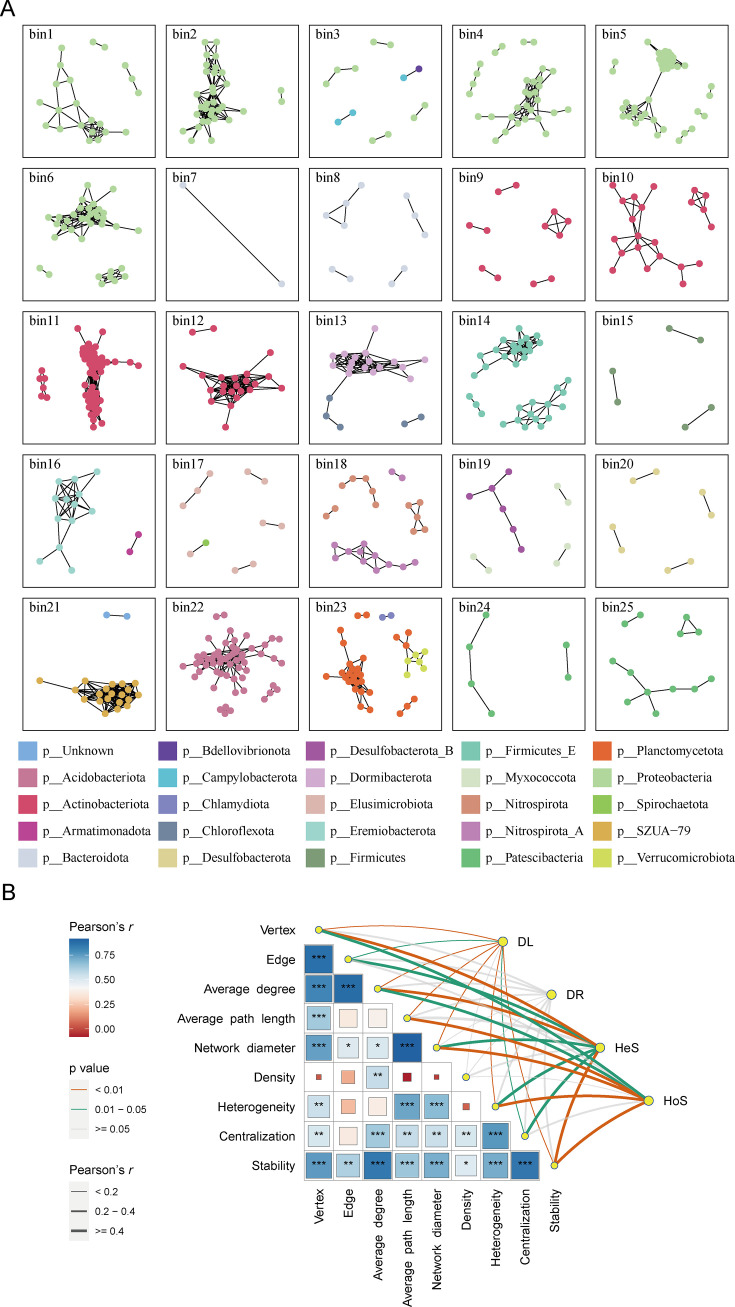
Co-occurrence networks of phylogenetic bins (**A**) and the associations between network properties and relative importance of ecological processes shaping the phylogenetic group assembly (**B**).

We constructed an interaction network for each phylogenetic bin to investigate how network properties drive phylogenetic bin assemblages (Fig. 3A). Our results showed that the interaction network properties of phylogenetic bins have a significant impact on ecological processes including dispersal limitation, heterogeneous selection, and homogeneous selection (Fig. 3B). Regarding stochastic processes, we observed a significant decrease in the relative importance of dispersal limitation with increases in network complexity, as measured by the number of vertices and edges, network diameter, average degree, average path length, heterogeneity, and network stability (Fig. S2). In turn, drift appeared to be unaffected by changes in these network properties. As for deterministic processes, we found that both heterogeneous selection and homogeneous selection have significantly positive correlations with the number of vertices, network diameter, average degree, heterogeneity, and network stability (all *P* < 0.05; Figure S3 and S4). Further random forest regression analysis showed that the number of vertices and edges was the most important driver of dispersal limitation and homogeneous selection. Additionally, average path length and network stability play a primary role in drift and heterogeneous selection, respectively.

At community level, we found that homogeneous selection significantly associated with two network properties of density (R^2^ = 0.155, *P* < 0.001; Table S3) and heterogeneity (R^2^ = 0.103, *P* < 0.001; Table S3), while drift significantly related to the number of vertices (R^2^ = 0.129, *P* < 0.001; Table S3) and heterogeneity (R^2^ = 0.112, *P* < 0.001; Table S3). These results indicated that ecological processes might play a different role in shaping network structure at phylogenetic bins and overall community.

### Orthologous genes associated with phylogenetic bin assemblage

**Fig 4:**
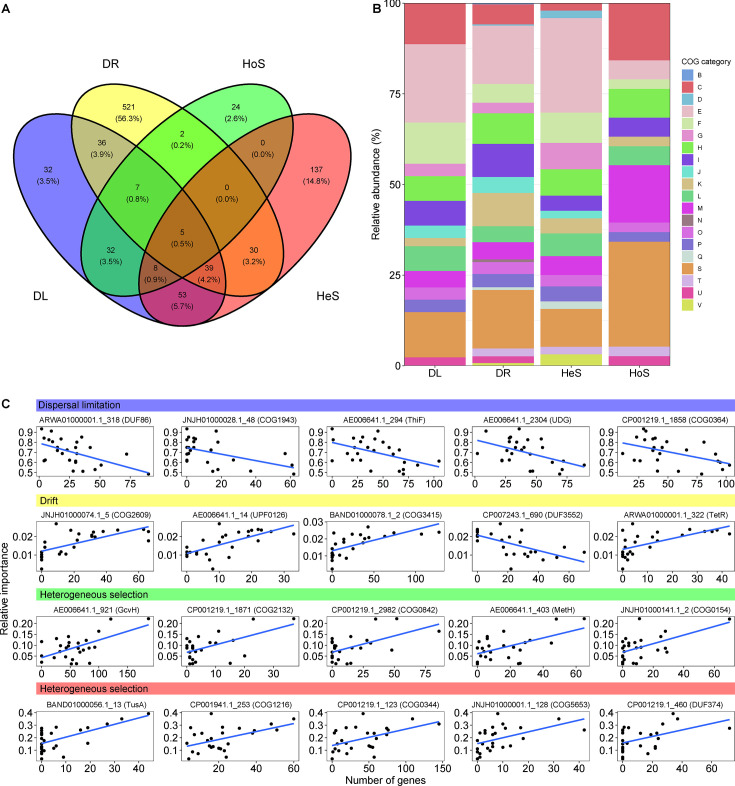
Functional genes constraining ecological processes shaping the phylogenetic group assembly. Venn diagram analysis of the genes across various ecological processes (**A**). Composition of COG function categories (**B**) and the linear relationships between relative importance of ecological processes and its top five most relevant genes (C).

Genes in metagenome-assembled genomes (MAGs) were clustered into 2,357 orthologous genes (OGs), of which 926 were significantly associated with ecological processes within phylogenetic assemblages. Specifically, drift was significantly affected by 640 OGs, followed by heterogeneous selection (272), dispersal limitation (212), and homogeneous selection (78). Results of Venn diagram analysis showed that only 5 OGs (AE006641.1_1014, AE006641.1_1013, AE006641.1_1015, AE006641.1_2111, and CP004145.1_1513) were significantly related to all four ecological processes (Fig. 4A), but these could not be annotated with known functions. Furthermore, we found that 53.5% of OGs (495) associated with ecological processes could be successfully aligned with the clusters of orthologous groups (COG) database (Fig. 4B). Ten COG categories of OGs were consistently related to all four ecological processes, including energy production and conversion (C); amino acid transport and metabolism (E); nucleotide transport and metabolism (F); coenzyme transport and metabolism (H); lipid transport and metabolism (I); transcription (K); replication, recombination, and repair (L); cell wall/membrane/envelope biogenesis (M); posttranslational modification, protein turnover, chaperones (O); and inorganic ion transport and metabolism (*P*). Specifically, only the drift process was significantly affected by chromatin structure and dynamics (B) and cell motility (N). We found that dispersal limitation was mostly correlated with the gene (ARWA01000001.1_318) encoding the DUF86 protein (Pearson’s *r* = −0.538, *P* = 0.005; Fig. 4C), and drift was mostly related to the gene (JNJH01000074.1_5) encoding the pyruvate dehydrogenase (*r* = −0.669, *P* < 0.001; Fig. 4C). As for selection processes, the relative importance of heterogeneous selection and homogeneous selection had the strongest correlation with the OG number of *gcvH* (AE006641.1_921; *r* = −0.580, *P* = 0.002; Fig. 4C) and *tusA* (BAND01000056.1_13; *r* = 0.636, *P* < 0.001; Fig. 4C), respectively.

### Influences of viral infection on ecological processes in phylogenetic bin assemblage

**Fig 5:**
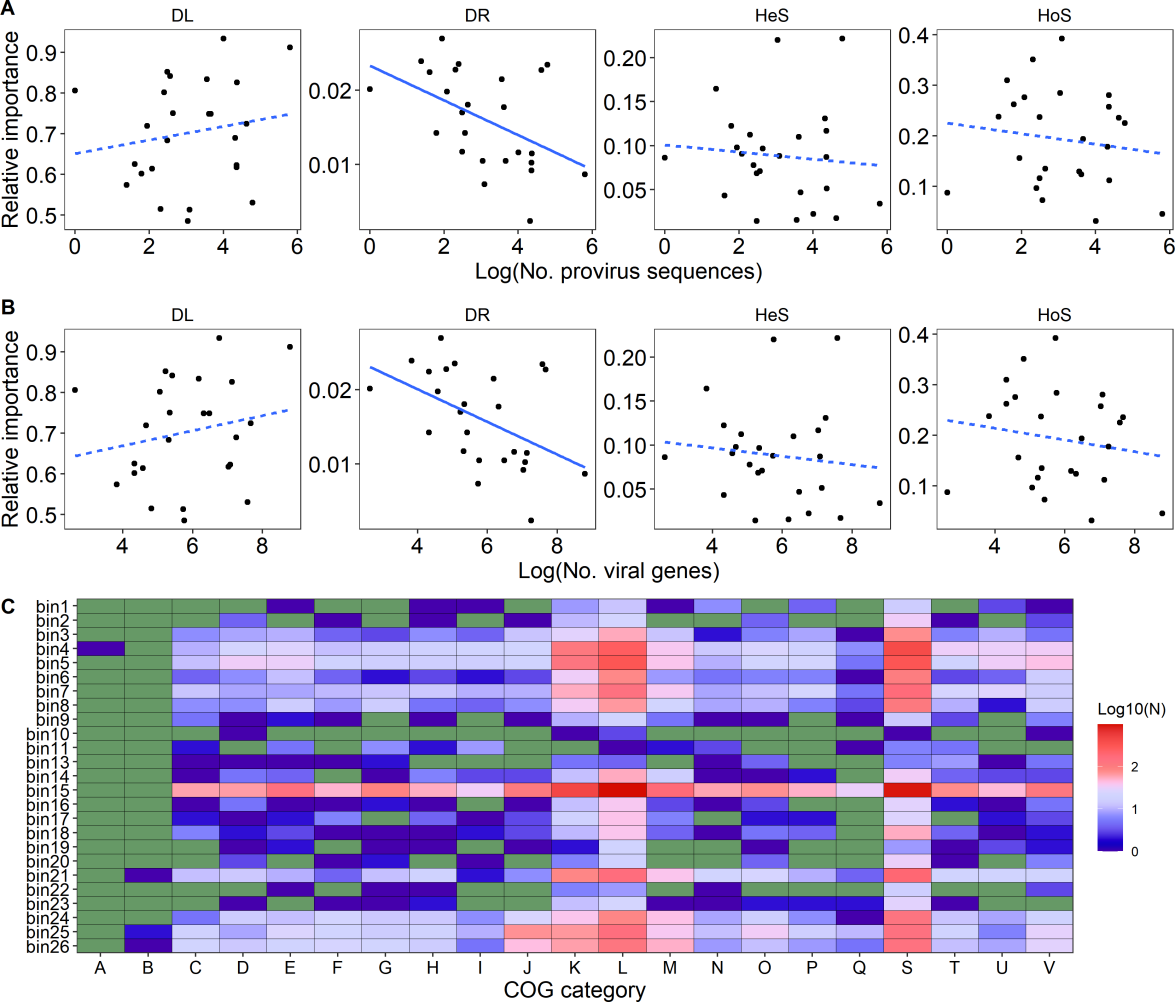
Influences of viral infections on phylogenetic bin assembly. Linear relationships between relative importance of ecological processes and log-transformed number of proviruses (**A**) or their genes in phylogenetic bins (**B**). Distribution of COG function categories for viral genes in phylogenetic bins (**C**).

A total of 1,511 provirus sequences were identified from 506 AMD microbial genomes. We found that the distribution of provirus sequences in phylogenetic bins followed the exponential distribution (*P* < 0.001, adjusted R^2^ = 0.945; Fig. S5). Specifically, viral infection mostly occurred in phylogenetic bin15 (331 provirus sequences), followed by bin5 (120) and bin4 (102), while bin10 was the least (1). Our results showed that the log-transformed number of proviruses significantly decreased the relative importance of drift in phylogenetic bin assemblages (*r* = −0.473, *P* = 0.017; Fig. 5A), as well as viral genes (*r* = −0.478, *P* = 0.016; Fig. 5B). We found that nestedness of host–virus network in phylogenetic bins had a significantly (*P* = 0.049) negative relationship with the relative importance of drift process, while modularity of that had no significant (*P* > 0.050) influence on ecological processes in phylogenetic bin assemblages. Furthermore, 64.2% of these viral genes were successfully assigned to COG function categories, most of which were widely distributed in phylogenetic bins (Fig. 5C; Fig. S6) such as replication, recombination and repair (L), transcription (K), and coenzyme transport and metabolism (H). We also found that two key COG function categories significantly decreased the relative importance of drift, including L (*r* = −0.434, *P* = 0.030) and H (*r* = −0.478, *P* = 0.024). Specifically, the number of viral genes involved in As resistance in phylogenetic bins had a significantly negative association with the relative importance of drift (R^2^ = 0.258, *P* = 0.044; Table S4).

## DISCUSSION

### Niche positions in environmental factors regulate determinism and stochasticity in microbial community assembly

Deviations between environmental conditions and relevant niche optima impose abiotic stress on species growth, which could lower the importance of stochastic processes in community assembly (1). Environmental factors often serve as the balancing factor between stochastic and deterministic processes. For example, bacterial community assembly in wastewater treatment plants was dominated by deterministic processes when the temperature was 30°C, while it became stochastic processes when the temperature was over 30°C (5). Similarly, extreme soil pH exerts strong selective pressures on bacterial community assembly toward phylogenetic overclustering, while near-neutral pH results in a similar phylogenetic structure of community compared with expectations from the null model (4). These results indicate that niche position of population along environmental gradient could regulate stochasticity in community assembly. Intriguingly, our results provide evidence that the primary process of dispersal limitation in AMD phylogenetic bins was dramatically weakened by increasing niche position of temperature (28.7~32.9°C). Consistently, species with niche position at high temperature were constrained more by environmental filtering than diversification during microbial community assembly in hot springs (67). Moreover, we found that the niche position of DO also significantly influenced ecological drift. Specifically, bin20 consisting of Bacillus members has the highest niche position of DO, and its assembly was dominated by dispersal limitation (85%). A higher niche position in oxygen means more terminal oxidase in electron transport system (68), which significantly improves bacterial growth and their competitiveness (69). Consequently, competitive exclusion by Bacillus was frequently observed in suppression of other species growth and competitiveness (69), and further limits dispersal processes in community assembly. Taken together, species niche position of environmental factors could regulate deterministic and stochastic processes in microbial community assembly.

### Dispersal limitation negatively associated with network stability

Ecological processes play a key role in the assembly of species interaction network, and their relative importance shifts with network properties as construction proceeds. In trophic interaction networks, niche-based processes prevail over neutral processes in plant–herbivorous insect networks as community succession occurs toward the next steady state after fire disturbance, indicated by increasing specialization and the proportion of selective species (14). Consistently, the partitioning of endophytic microbiome in interaction network was influenced by species niche position in nutrients, which results in a niche-overlap network structure (70). Our results showed that co-occurrence network stability of phylogenetic bins negatively correlated with the relative importance of dispersal limitation, while it positively correlated with that of homogeneous selection or heterogeneous selection. Similarly, a higher network stability is accompanied by less dispersal limitation and more selection processes in marine eukaryotic community assemblage (71). This implies that changes in network structure could be mediated by dispersal limitation (72). The absence of dispersal limitation in more stable networks promotes more species coexisting in local communities, in which immigrating species may be excluded by fierce competition from similarly resident species with similar niche positions (73), which would shift the primary processes from dispersal limitation toward environmental selection, along with more intense species competitions in community assemblage. Meanwhile, dispersal weakened the role of keystone species in contributing to network complexity and decentralizing interaction networks (72), which results in increasing network stability. This association between dispersal limitation and network stability might have important implications for studying the stabilizing effect of space on microbial community.

### Coupling microbial community assembly to potential metabolic processes

Metabolic processes govern microbial behaviors and fundamentally drive microbial community assembly. In stochastic processes, it is more likely to be associated with broad function because species are ecologically similar. For instance, stochasticity in bacterial community assembly was strengthened by those genes involved in broad functions, including glycolysis/gluconeogenesis and tricarboxylic acid (TCA) cycle (22). We also found that the dominant process of dispersal limitation was negatively related to the increasing abundance of genes encoding the DUF86 protein, which is a conserved and abundant prokaryotic toxin component in the type II toxin-antitoxin (TA) system. This system has the potential to mediate a stochastic switch in bacterial cells to a slower growth rate, thereby enhancing their tolerance when exposed to environmental stress (39). This adaptation reduces the influence of environmental filtering while amplifying biotic filtering during community assembly. Consequently, this may generally decrease the stochasticity observed in the assembly of AMD microbial community. Overall, our results showed that ecological drift was constrained by most genes, although it was the least important process in AMD microbial community assembly. The proportion of genes encoding a transcription regulator associated with drift process was higher than that of other processes. Specifically, most of these transcription regulators consist of helix-turn-helix domain and belong to various families such as TetR (74), MerR (75), PadR (76), and LysR (77) (Table S2). These gene families were widely harbored by prokaryotes and were involved in the regulation of processes responding rapidly to environmental changes, including motility, biofilm formation, and stress responses to toxic chemicals, metal ions, and endogenous metabolites. These results revealed that stochastic processes may be greatly constrained by ubiquitous genes responding to environmental stress in microbial community assembly.

In deterministic processes, microbial species are selected by the abiotic environment and both antagonistic and synergistic interactions (3), resulting from their metabolic advantages in environmental fitness and competitiveness. For example, the soil microbial community exhibited the highest activity of carbon metabolism in organic carbon mineralization under maximum environmental selection but minimum dispersal (23). Similarly, the relative importance of deterministic processes was raised by increasing abundance of specialized functions, including sulfur metabolism, nitrogen metabolism, and methane metabolism (22). On the one hand, we found that heterogeneous selection process in phylogenetic group assembly was mostly constrained by genes involved in amino acid transport and metabolism. Specifically, HeS showed the highest positive correlation with abundance of *irrE* gene (AE006641.1_656; *P* = 0.026, adjusted R^2^ = 0.157), which was a global regulator to enhance stress tolerance, such as to high temperatures (78) and heavy metal (79, 80). On the other hand, our results showed that homogeneous selection process was primarily influenced by genes participating in cell wall/membrane/envelope biogenesis. Sediment microbial community dominated by HoS process contained the most abundant genes encoding glycosyltransferase in carbohydrate degradation and metabolism (81). We found that HoS was positively related to the gene abundance belonging to glycosyltransferase family 2, which promotes growth rate (82) but decreases tolerance to osmotic stress and cell wall stress. This indicates that glycosyl transferase may contribute to homogeneous selection through competition exclusion rather than abiotic filtering. These results indicated that deterministic processes were more likely imposed by specialized genes involved in improving fitness and competitiveness in microbial community assembly.

### Modulator of viruses between deterministic and stochastic processes

Viral infections on host species could bidirectionally regulate the stochasticity in microbial community assembly. For example, viral predation counterbalances homogeneous selection of hypersaline stress and drives microbial community toward undominated processes in fractured shale ecosystems (83). Viruses also constrained the dispersal processes of AMD microbial community assembly (37). This may be attributed to their auxiliary metabolic genes for host environmental adaptation, such as to low pH (37) and heavy metal (84), which offset abiotic selections. These results reveal that viral infection could enhance stochasticity in community assembly. Conversely, viral infection facilitates fitness or competitiveness in a range of host species and increases the importance of deterministic processes. For instance, stochastic assembly of virus community promotes the spread of resistance genes to organochlorine pesticide among host species, which was accompanied by a decrease in stochasticity in soil microbial community assembly (85). In this case, homogeneous selection imposed on microbial community assembly would shift to heterogeneous selection. Our results showed that increasing viral infection weakens the relative importance of ecological drift in phylogenetic bin assemblages as well as affects some viral genes involved in replication, recombination, and repair (L), and coenzyme transport and metabolism (H). Most of these genes display a broad and nonspecific response to stress, such as DNA methylase and recombinase (86). This expands the ecological difference of species in fitness and thus impedes drift in community assembly. These results reveal that viruses serving as modulators could bidirectionally control stochasticity in shaping communities.

### Conclusion

This study elucidates the balance between stochastic and deterministic processes in microbial community assembly within acid mine drainage. Our findings underscore that dispersal limitation is the predominant stochastic force, influenced significantly by niche positions of environmental variables, such as temperature and dissolved oxygen levels. Conversely, deterministic processes, primarily homogeneous and heterogeneous selections, are substantially shaped by microbial metabolic functions and network interactions. The relative importance of these deterministic processes is significantly associated with network properties such as stability and complexity. Furthermore, the impact of viral infections introduces an additional layer of complexity in community assembly. Our results suggest that viral dynamics can modulate the balance between stochastic and deterministic processes by influencing the genomic landscape, particularly through genes involved in replication, recombination, and repair. In summary, our study provides a comprehensive overview of the forces driving microbial community assembly in acid mine drainage. These findings not only advance our understanding of microbial ecology but also highlight the complex interdependencies that define ecological community in harsh environments.

## Data Availability

Our metagenomic sequence data can be accessed from the National Omics Data Encyclopedia (NODE) database under accession numbers OEP001841, OEP002738, and OEP001327. Other metagenomic sequence data used in this study are publicly available from the National Center for Biotechnology Information (NCBI) according to the accession numbers listed in Table S1.

## SUPPLEMENTAL MATERIAL

Supplemental material aem.01028-24-s0001.docx Tables S2 to S4; Figures S1 to S6.

Table S1 aem.01028-24-s0002.txt Metadata for metagenomic samples.

ASM does not own the copyrights to Supplemental Material that may be linked to, or accessed through, an article. The authors have granted ASM a non-exclusive, world-wide license to publish the Supplemental Material files. Please contact the corresponding author directly for reuse.
