# Supplementary material for: Ecological features of microbial community linked to stochastic and deterministic assembly processes in acid mine drainage
Source: Appl Environ Microbiol. 2024 Dec 16;91(1):e01028-24. doi: 10.1128/aem.01028-24 (PMC11784436; doi:10.1128/aem.01028-24)
Supplement: Supplemental material — Tables S2 to S4; Figures S1 to S6. [file aem.01028-24-s0001.docx]

**Supplementary Information for**

Ecological features of microbial community linked to stochastic and deterministic assembly processes in acid mine drainage

Zhenghua Liu^1^, Chengying Jiang^2^, Zhuzhong Yin^1^, Ibrahim Ahmed Ibrahim^3^, Teng Zhang^1^, Jing Wen^1^, Lei Zhou^4^, Guoping Jiang^4^, Liangzhi Li^1^, Zhendong Yang^5^, Ye Huang^2^, Zhaoyue Yang^1^, Yabing Gu^1^, Delong Meng^1^, Huaqun Yin^1*^

^1^ School of Minerals Processing and Bioengineering, Key Laboratory of Biometallurgy of Ministry of Education, Central South University, Changsha 410083, China

^2^ State Key Laboratory of Microbial Resources, Institute of Microbiology, Chinese Academy of Sciences, Beijing 100101, China

^3^Central Metallurgical Research and Development Institute, Cairo 11421, Egypt

^4^ Hebei Key Laboratory of Highly Efficient Exploitation and Utilization of Radioactive Mineral Resources

^5^ School of Architecture and Civil Engineering, Chengdu University, Chengdu, 610106, Sichuan, China

**Running title: The forces driving AMD community assembly**

**The file includes:**

Supplementary Table (S2-S4)

Supplementary Figures (S1–S6)

Table S2. Linear relationships between the relative importance of drift and the number of genes encoding transcriptional regulator.

| OGID | Slop | P value | Adjusted R^2^ | Function |
| --- | --- | --- | --- | --- |
| ARWA01000001.1_322 | 0.00029 | 0.00061 | 0.36734 | WHG domain |
| CP001219.1_356 | 0.00007 | 0.00394 | 0.26840 | transcriptional regulator, MerR |
| CP001219.1_2821 | 0.00015 | 0.00582 | 0.24613 | Belongs to the UPF0301 (AlgH) family |
| BAND01000120.1_36 | 0.00021 | 0.00661 | 0.23877 | HxlR-like helix-turn-helix |
| CP001219.1_535 | 0.00014 | 0.00927 | 0.21887 | Promotes RNA polymerase assembly |
| CP001219.1_588 | 0.00024 | 0.01590 | 0.18649 | Helix-turn-helix domain |
| ARWA01000001.1_3923 | 0.00014 | 0.01691 | 0.18276 | Bacterial regulatory proteins, tetR family |
| AE006641.1_41 | 0.00023 | 0.01924 | 0.17484 | PFAM transcriptional regulator PadR family protein |
| ARWA01000001.1_428 | 0.00030 | 0.01931 | 0.17464 | Helix-turn-helix |
| CP001219.1_1826 | 0.00043 | 0.02234 | 0.16568 | AsnC-type helix-turn-helix domain |
| JQMQ01000004.1_478 | 0.00016 | 0.02879 | 0.14996 | Bacterial transcriptional repressor C-terminal |
| BAND01000093.1_15 | 0.00012 | 0.02912 | 0.14924 | phosphorelay signal transduction system |
| AE006641.1_83 | 0.00004 | 0.02970 | 0.14802 | TIGRFAM ribosomal-protein-alanine acetyltransferase |
| JQMQ01000004.1_556 | 0.00021 | 0.03098 | 0.14538 | Sigma-70, region 4 |
| AFOH01000038.1_7 | 0.00018 | 0.03152 | 0.14430 | Helix-turn-helix XRE-family like proteins |
| CP001219.1_1970 | 0.00017 | 0.03549 | 0.13690 | Cro/C1-type HTH DNA-binding domain |
| CP001219.1_131 | 0.00007 | 0.03588 | 0.13621 | Transcriptional regulator, LysR |
| CP001219.1_287 | 0.00012 | 0.03722 | 0.13391 | Negatively regulates transcription of bacterial ribonucleotide reductase nrd genes |
| ARWA01000001.1_2689 | 0.00019 | 0.03951 | 0.13017 | Bacterial regulatory proteins, tetR family |
| CP001219.1_90 | 0.00008 | 0.04189 | 0.12649 | TIGRFAM Addiction module antidote protein, HigA |
| CP001219.1_37 | 0.00006 | 0.04448 | 0.12272 | LysR substrate binding domain |
| JFHO01000224.1_1 | 0.00019 | 0.04587 | 0.12079 | helix_turn_helix multiple antibiotic resistance protein |
| JFHO01000119.1_17 | 0.00009 | 0.04589 | 0.12076 | helix_turn_helix multiple antibiotic resistance protein |
| JQMQ01000005.1_837 | 0.00012 | 0.04647 | 0.11996 | RNA polymerase Rpb6 |
| ARWA01000001.1_261 | 0.00010 | 0.04721 | 0.11897 | AefR-like transcriptional repressor, C-terminal region |

Table S3. Linear relationships between the relative importance of ecological processes and the number of viral genes involving in metal resistance.

| Ecological Process | Properties | Slope | P | Std. Error | Adjusted R^2^ |
| --- | --- | --- | --- | --- | --- |
| HeS | Vertex | 130.949 | 0.180 | 96.924 | 0.008 |
| HoS | Vertex | -131.727 | 0.073 | 72.620 | 0.023 |
| DL | Vertex | 31.977 | 0.653 | 70.894 | -0.008 |
| DR | Vertex | 3264.898 | <0.001 | 825.296 | 0.129 |
| HeS | Edge | 486.511 | 0.636 | 1025.190 | -0.008 |
| HoS | Edge | -574.651 | 0.459 | 772.443 | -0.005 |
| DL | Edge | 80.541 | 0.914 | 744.553 | -0.010 |
| DR | Edge | 30485.193 | 0.001 | 8801.785 | 0.100 |
| HeS | Average degree | -2.398 | 0.358 | 2.598 | -0.001 |
| HoS | Average degree | 0.400 | 0.839 | 1.969 | -0.010 |
| DL | Average degree | 0.561 | 0.768 | 1.892 | -0.009 |
| DR | Average degree | 52.006 | 0.027 | 23.120 | 0.039 |
| HeS | Average path length | 0.493 | 0.077 | 0.276 | 0.022 |
| HoS | Average path length | -0.189 | 0.371 | 0.210 | -0.002 |
| DL | Average path length | -0.109 | 0.592 | 0.203 | -0.007 |
| DR | Average path length | 3.827 | 0.131 | 2.515 | 0.013 |
| HeS | Network diameter | 1.535 | 0.444 | 1.999 | -0.004 |
| HoS | Network diameter | 0.682 | 0.653 | 1.511 | -0.008 |
| DL | Network diameter | -1.514 | 0.298 | 1.446 | 0.001 |
| DR | Network diameter | 11.887 | 0.515 | 18.174 | -0.006 |
| HeS | Clustering coefficient | -0.008 | 0.813 | 0.034 | -0.010 |
| HoS | Clustering coefficient | 0.013 | 0.623 | 0.025 | -0.008 |
| DL | Clustering coefficient | -0.003 | 0.898 | 0.024 | -0.010 |
| DR | Clustering coefficient | -0.668 | 0.028 | 0.299 | 0.039 |
| HeS | Density | -0.021 | 0.045 | 0.010 | 0.031 |
| HoS | Density | 0.032 | <0.001 | 0.007 | 0.155 |
| DL | Density | -0.017 | 0.026 | 0.007 | 0.040 |
| DR | Density | -0.247 | 0.008 | 0.092 | 0.059 |
| HeS | Heterogeneity | 0.136 | 0.050 | 0.068 | 0.029 |
| HoS | Heterogeneity | -0.174 | 0.001 | 0.050 | 0.103 |
| DL | Heterogeneity | 0.076 | 0.133 | 0.050 | 0.013 |
| DR | Heterogeneity | 2.181 | <0.001 | 0.593 | 0.112 |
| HeS | Centralization | -0.028 | 0.024 | 0.012 | 0.041 |
| HoS | Centralization | 0.027 | 0.003 | 0.009 | 0.078 |
| DL | Centralization | -0.009 | 0.302 | 0.009 | 0.001 |
| DR | Centralization | -0.220 | 0.049 | 0.110 | 0.029 |
| HeS | Stability | -0.030 | 0.178 | 0.022 | 0.008 |
| HoS | Stability | 0.011 | 0.534 | 0.017 | -0.006 |
| DL | Stability | 0.005 | 0.778 | 0.016 | -0.009 |
| DR | Stability | 0.243 | 0.236 | 0.204 | 0.004 |

Table S4. Linear relationships between the relative importance of ecological processes and the number of viral genes involving in metal resistance.

| Ecological processes | Metal | Slope | P | Std. Error | Adjusted R2 | N |
| --- | --- | --- | --- | --- | --- | --- |
| DL | As | 0.021 | 0.117 | 0.012 | 0.137 | 13 |
| DL | Cu | -0.007 | 0.697 | 0.017 | -0.103 | 10 |
| DL | Hg | 0.002 | 0.826 | 0.009 | -0.094 | 12 |
| DL | Ni | 0.010 | 0.473 | 0.014 | -0.056 | 9 |
| DL | W | 0.014 | 0.188 | 0.010 | 0.124 | 9 |
| **DR** | **As** | **-0.001** | **0.044** | **0.001** | **0.258** | **13** |
| DR | Cu | -0.001 | 0.381 | 0.001 | -0.016 | 10 |
| DR | Hg | 0.000 | 0.530 | 0.001 | -0.055 | 12 |
| DR | Ni | 0.000 | 0.853 | 0.001 | -0.137 | 9 |
| DR | W | 0.000 | 0.420 | 0.000 | -0.034 | 9 |
| HeS | As | -0.004 | 0.396 | 0.005 | -0.019 | 13 |
| HeS | Cu | 0.004 | 0.652 | 0.009 | -0.095 | 10 |
| HeS | Hg | -0.005 | 0.323 | 0.005 | 0.007 | 12 |
| HeS | Ni | -0.007 | 0.171 | 0.004 | 0.142 | 9 |
| HeS | W | -0.005 | 0.342 | 0.005 | 0.005 | 9 |
| HoS | As | -0.015 | 0.145 | 0.010 | 0.108 | 13 |
| HoS | Cu | 0.003 | 0.744 | 0.010 | -0.109 | 10 |
| HoS | Hg | 0.002 | 0.694 | 0.006 | -0.082 | 12 |
| HoS | Ni | -0.004 | 0.747 | 0.011 | -0.125 | 9 |
| HoS | W | -0.009 | 0.192 | 0.006 | 0.119 | 9 |


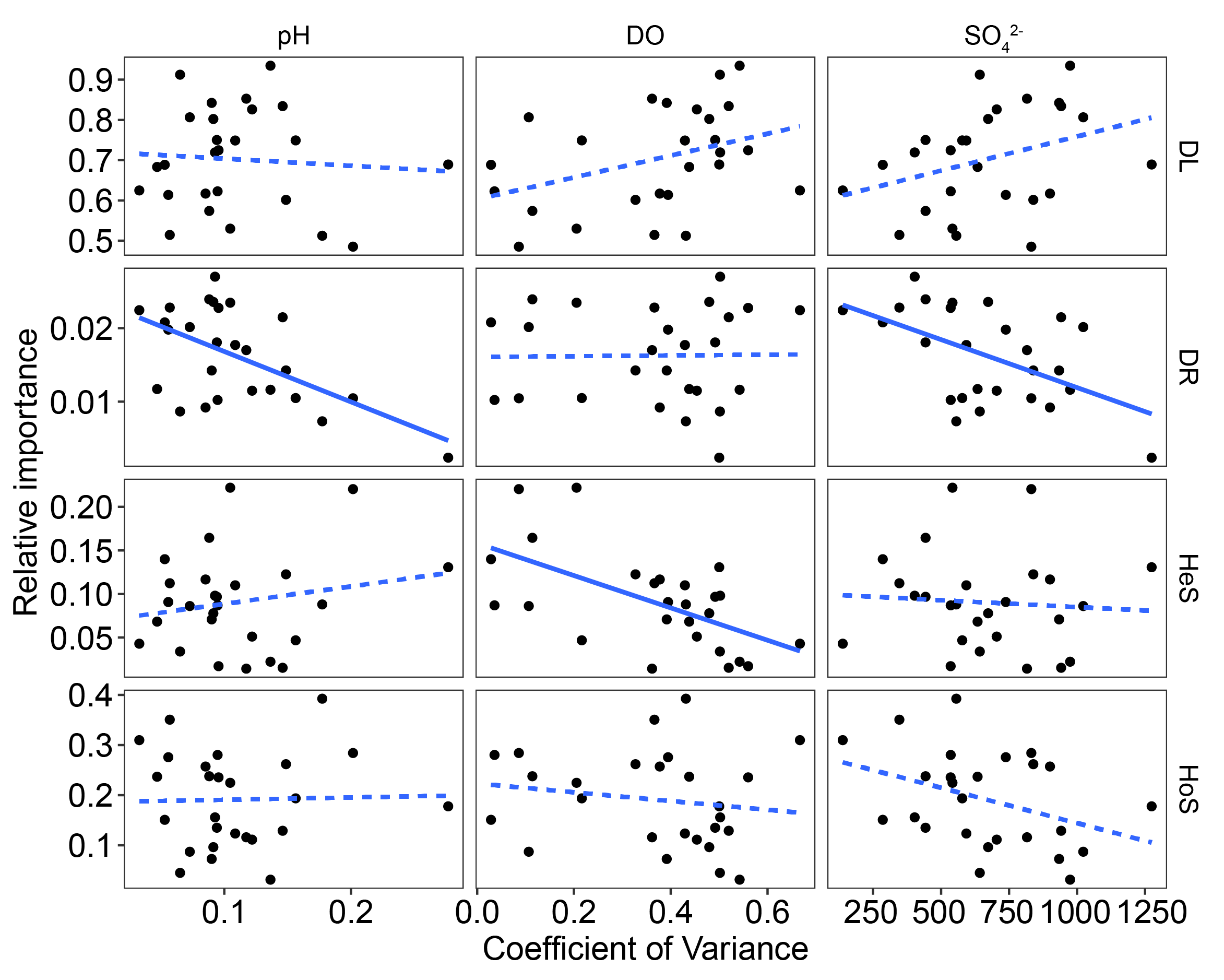


Figure S1. Linear relationships between the relative importance in ecological processes and coefficient of variance in environmental niche optima. Solid line: P < 0.05; Dashed line: P ≥ 0.05.


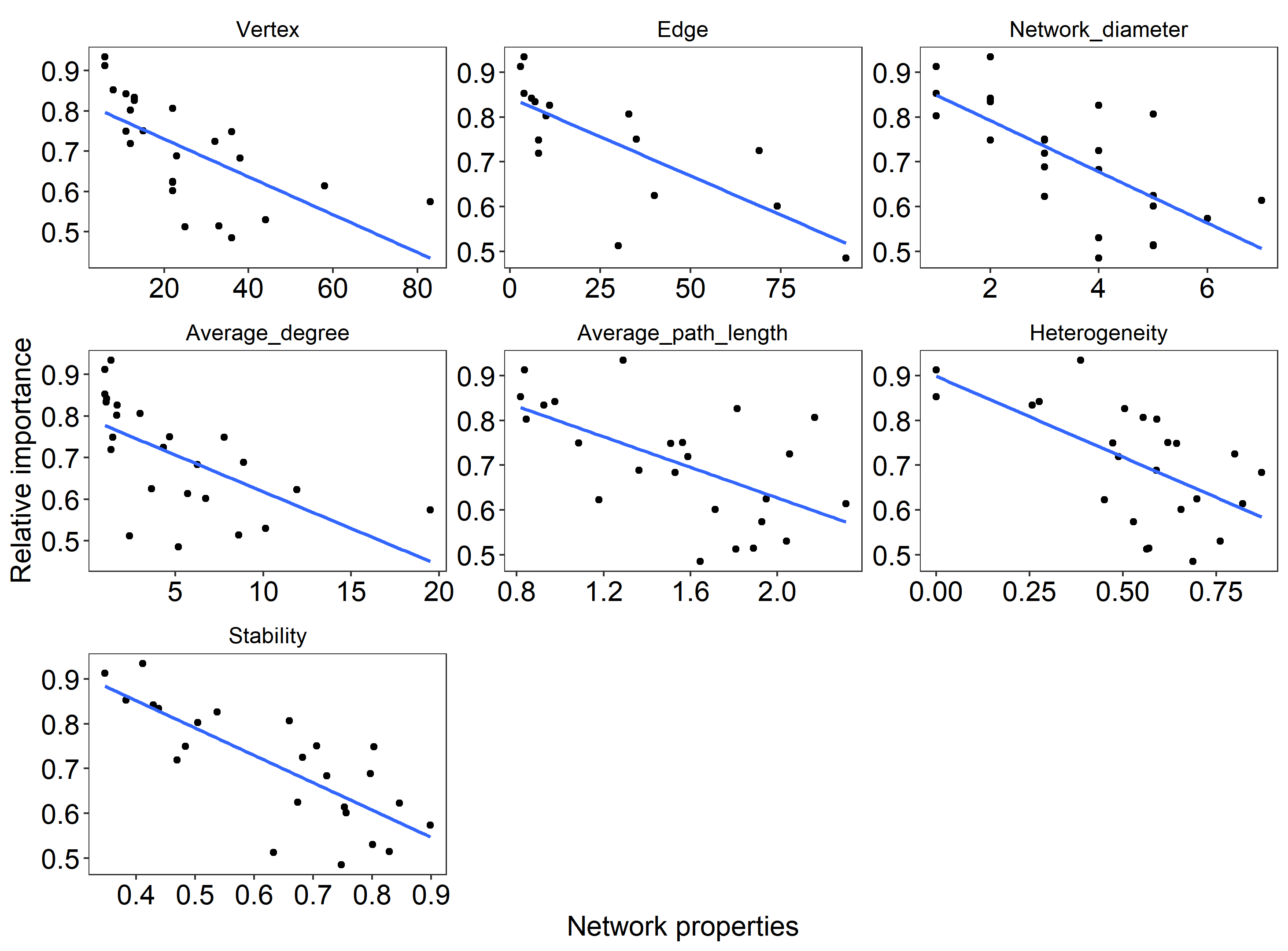


Figure S2. Significant linear relationships between the relative importance in dispersal limitation and network properties of phylogenetic bins.


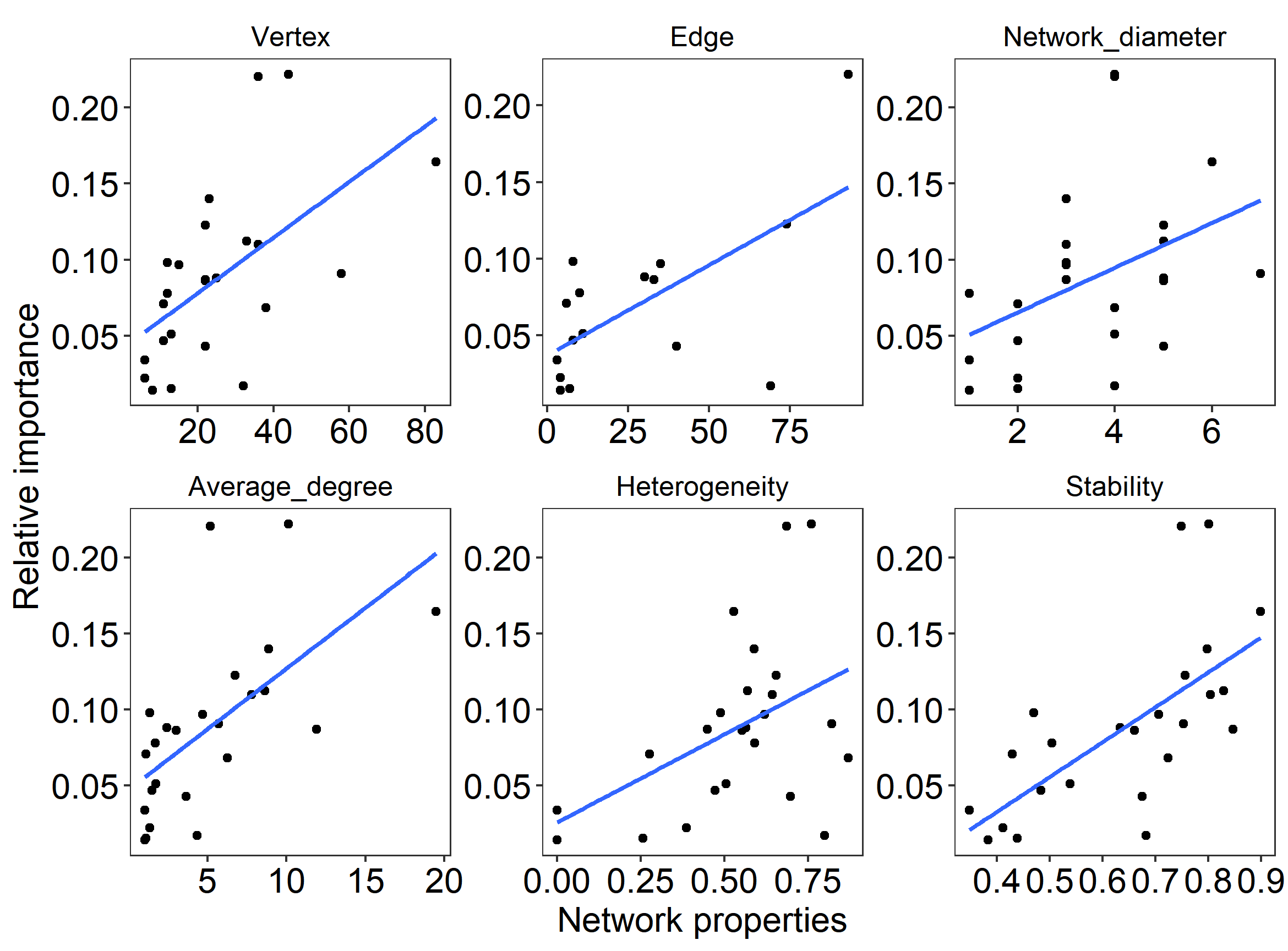


Figure S3. Significant linear relationships between the relative importance in heterogeneous selection and network properties of phylogenetic bins.


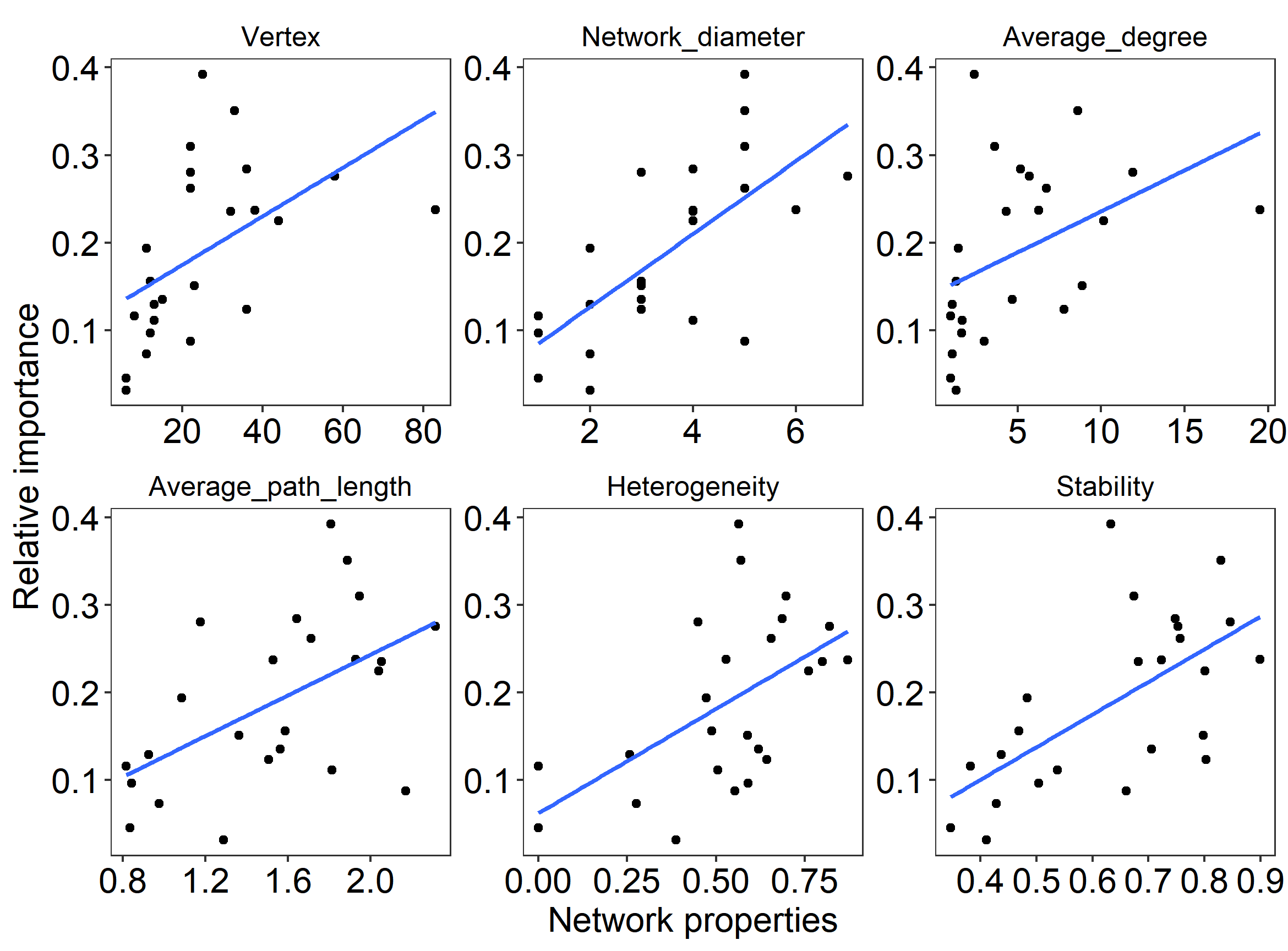


Figure S4. Significant linear relationships between the relative importance in homogeneous selection and network properties of phylogenetic bins.


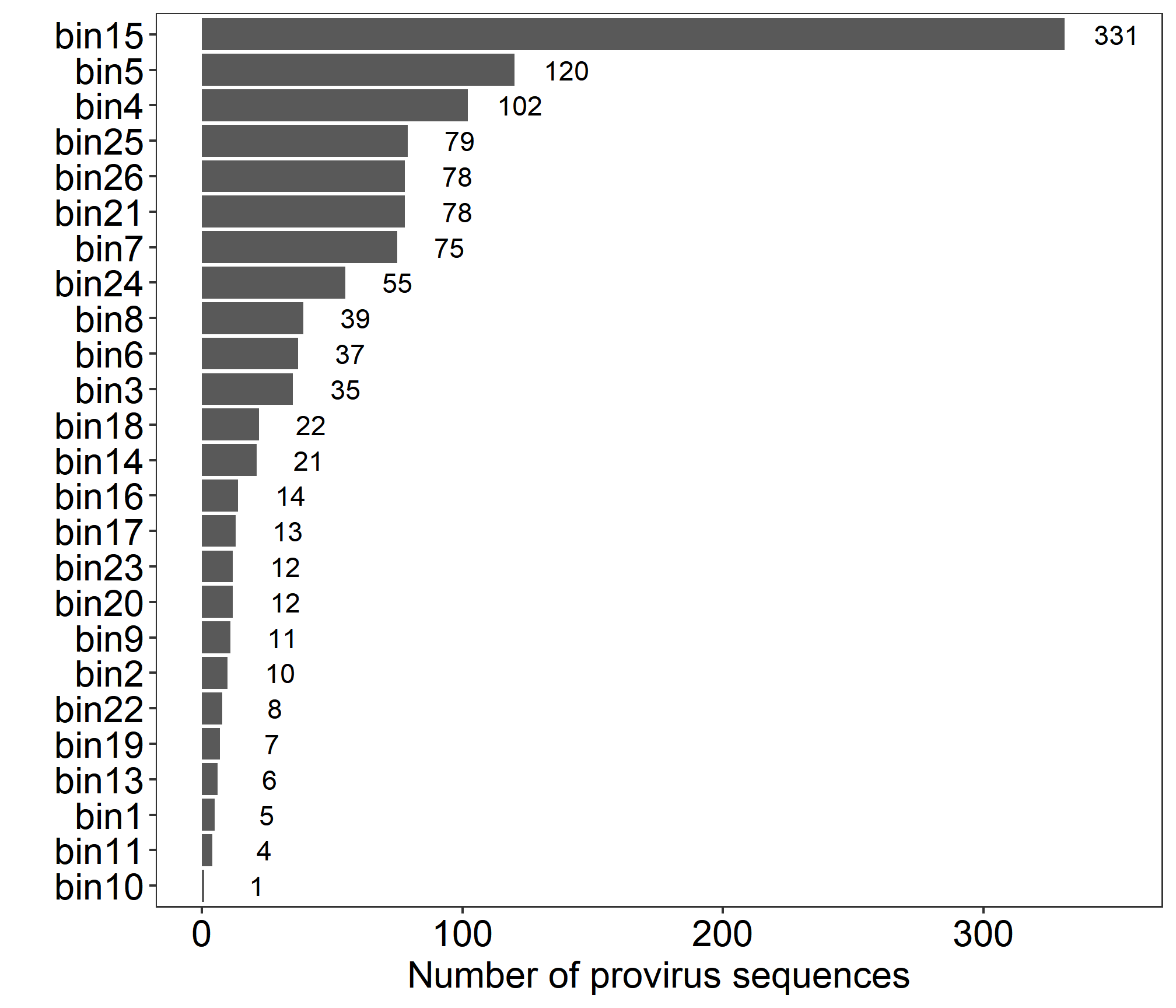


Figure S5. Number of provirus sequences across various phylogenetic bins.


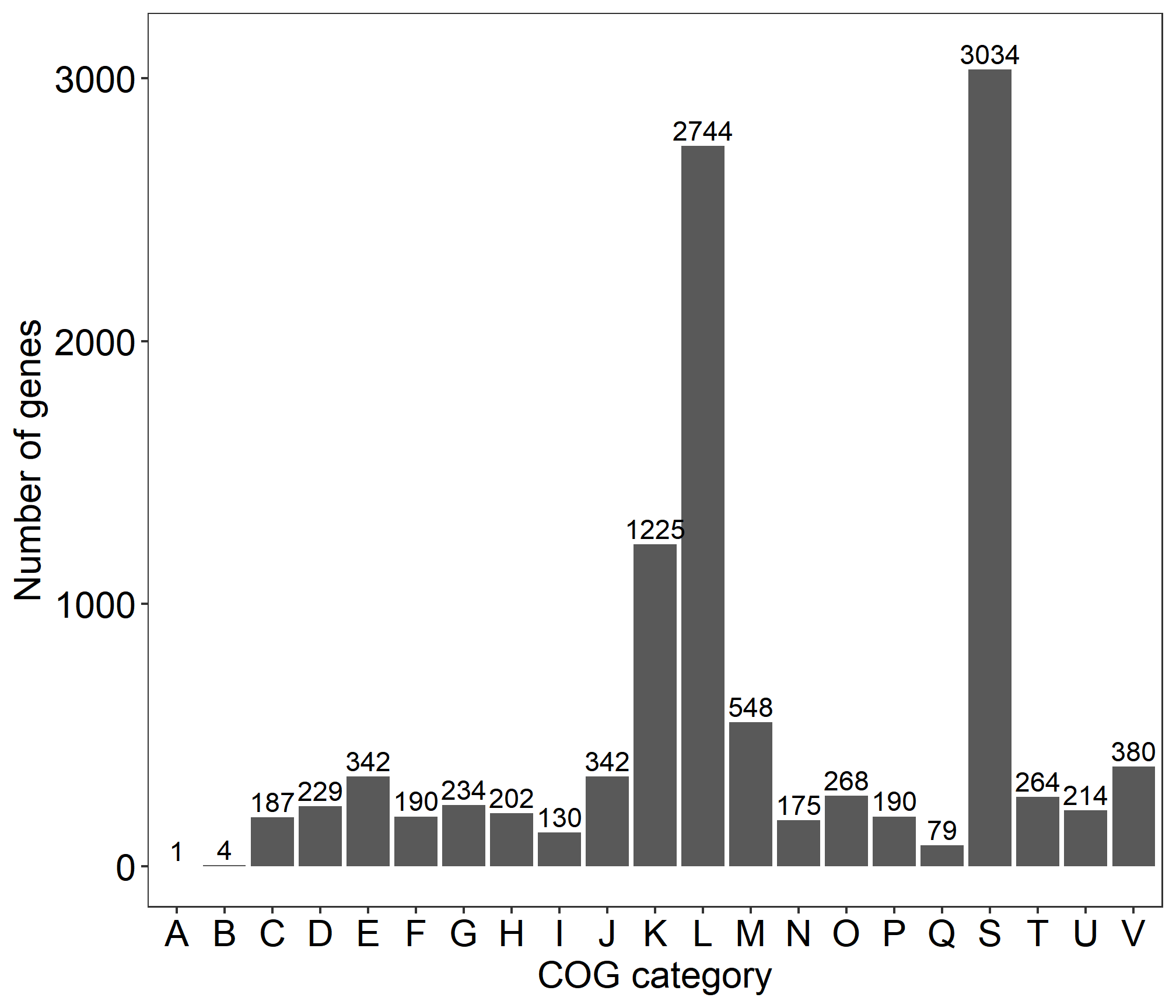


Figure S6. Number of viral genes assigned with COG function categories.
